# Supplementary figures and images for: Mutant EZH2 alters the epigenetic network and increases epigenetic heterogeneity in B cell lymphoma
Source: PLoS Biol. 2025 Jun 12;23(6):e3003191. doi: 10.1371/journal.pbio.3003191 (PMC12161531; doi:10.1371/journal.pbio.3003191)

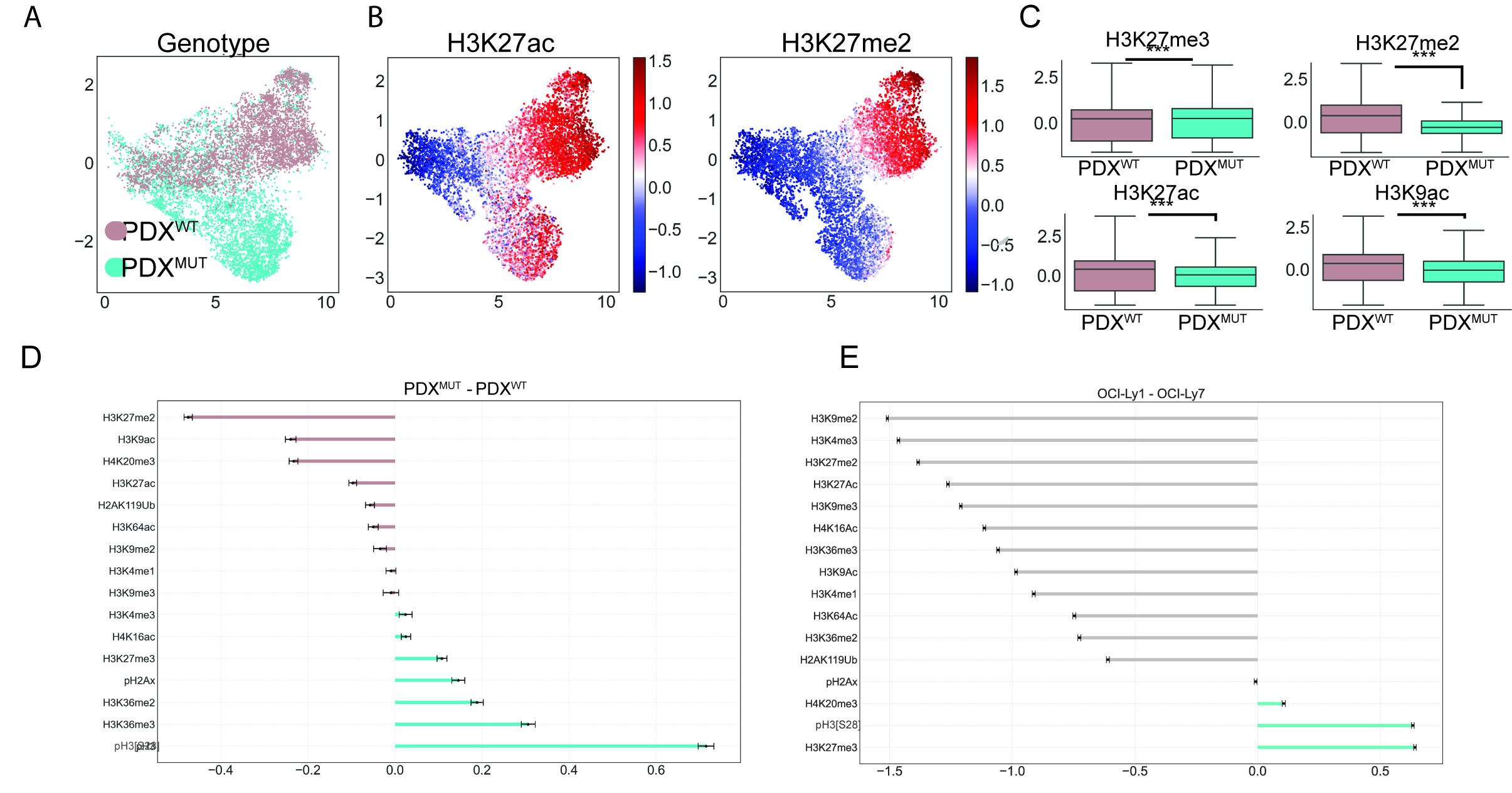

Supplement: S1 Fig — A. Two patient-derived xenografts; PDXWT which carries WT copies of EZH2 and KMT2D, and PDXMUT which carries the EZH2 Y646N mutation and biallelic loss of KMT2D, were analyzed by CyTOF. UMAP was performed based on all epigenetic marks measured, following scaling and normalization. Colors indicate the sample index. B. Scaled, normalized levels of the indicated histone modifications on the UMAP of the PDXs that is shown in A. C. Expression levels of the indicated modifications in PDXWT and PDXMUT, as measured by the CyTOF. P values were calculated by Welch’s t test. ***p value < 0.001. D. Mean differences between PDXWT and PDXMUT for the indicated histone modifications. The mean values for PDXWT were subtracted from PDXMUT. Uncertainties were estimated using bootstrapping. E. Mean differences between OCI-Ly7 and OCI-Ly1 for the indicated histone modifications. The mean values for OCI-Ly7 (WT) were subtracted from OCI-Ly1 (EZH2 Y646N). Uncertainties were estimated using bootstrapping. The data underlying this figure can be found in Raw data 1 at 10.17605/OSF.IO/NTGUX, under CyTOF folder. (TIF) [file pbio.3003191.s001.tif]

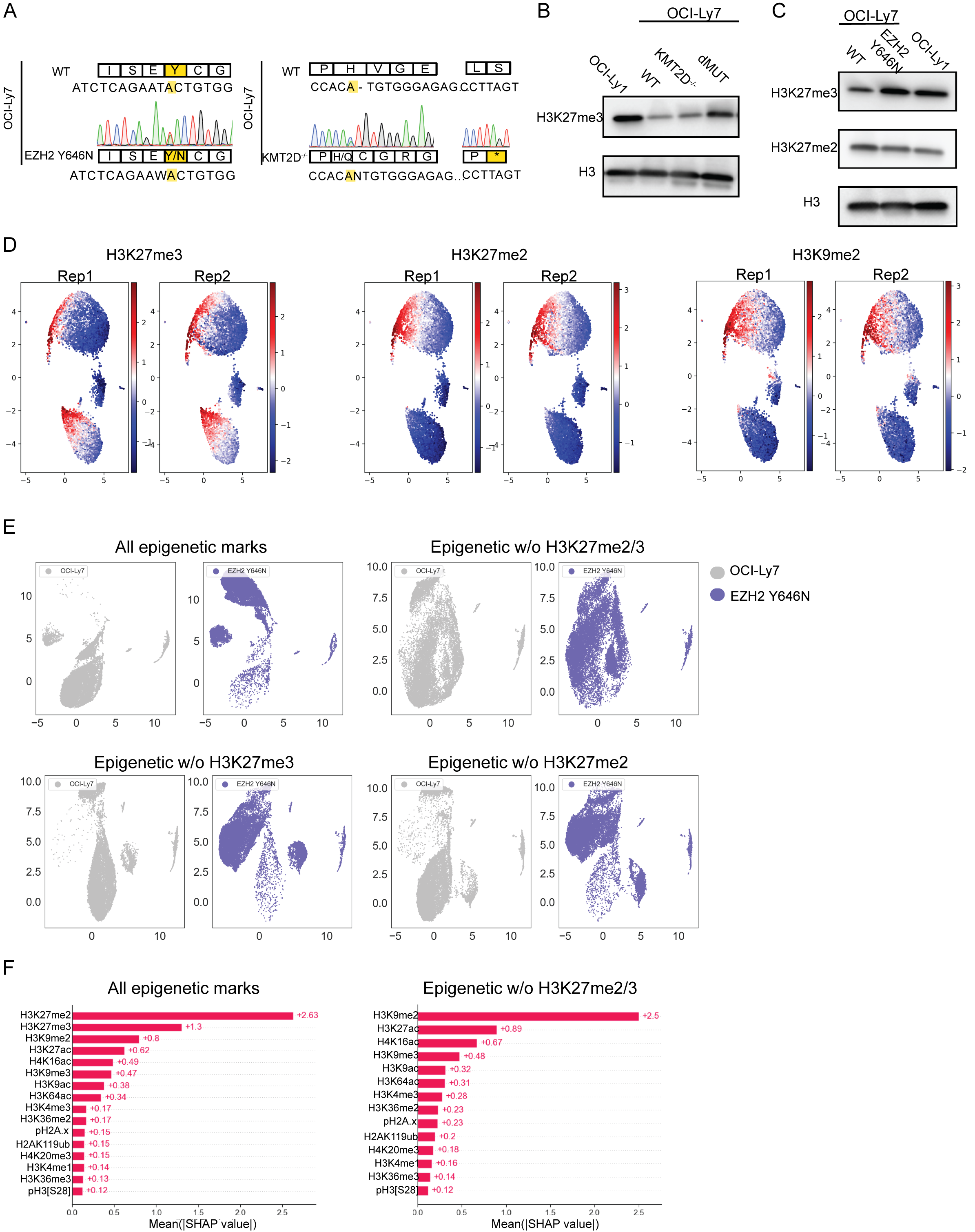

Supplement: S2 Fig — A. Left: Sequencing traces of Exon 16 of EZH2, indicating the heterozygous gain-of-function EZH2 Y646N mutation, generated by CRISPR-Cas9 genome editing, in OCI-Ly7 cells. Right: Sequencing traces of Exon 2 of KMT2D, indicating a biallelic insertion of A/C by CRISPR-Cas9 genome editing resulting in a premature stop codon, in OCI-Ly7 cells. B–C. Western blot analysis of the indicated modifications in the isogenic OCI-Ly7 WT cells and their counterparts carrying the EZH2 GOF mutation, KMT2D knockout, or a combination of both. Also shown are OCI-Ly1 cells expressing mutant-EZH2 and biallelic loss of KMT2D. Histone H3 represents loading control. D. OCI-Ly7 cells (WT), as well as their isogenic counterparts carrying mutant-EZH2 (EZH2 Y646N), biallelic knockout of KMT2D (KMT2D−/−), or a combination of both mutations in EZH2 and KMT2D (dMUT), were analyzed by CyTOF. UMAP was performed based on all epigenetic marks measured in two independent biological repeats, following scaling and normalization (see Fig 1E). Shown are the indicated modifications. E. OCI-Ly7 and EZH2 Y646N were analyzed by CyTOF, all epigenetic marks were scaled and normalized. UMAP was performed based on all epigenetic marks measured, all marks without H3K27me2/3, or all marks without either H3K27me3 or H3K27me2, as indicated. Colors indicate the sample index. F. SHAP analysis between OCI-Ly7 and EZH2 Y646N, indicating the epigenetic marks that contribute to the separation between the two cell lines. H3K27me2, H3K27me3, H3K9me2 and H3K27ac contribute most to the separation of the two cell lines. The data underlying this figure can be found in Raw data 1 at 10.17605/OSF.IO/NTGUX, under CyTOF folder and S1 Raw images. (TIF) [file pbio.3003191.s002.tif]

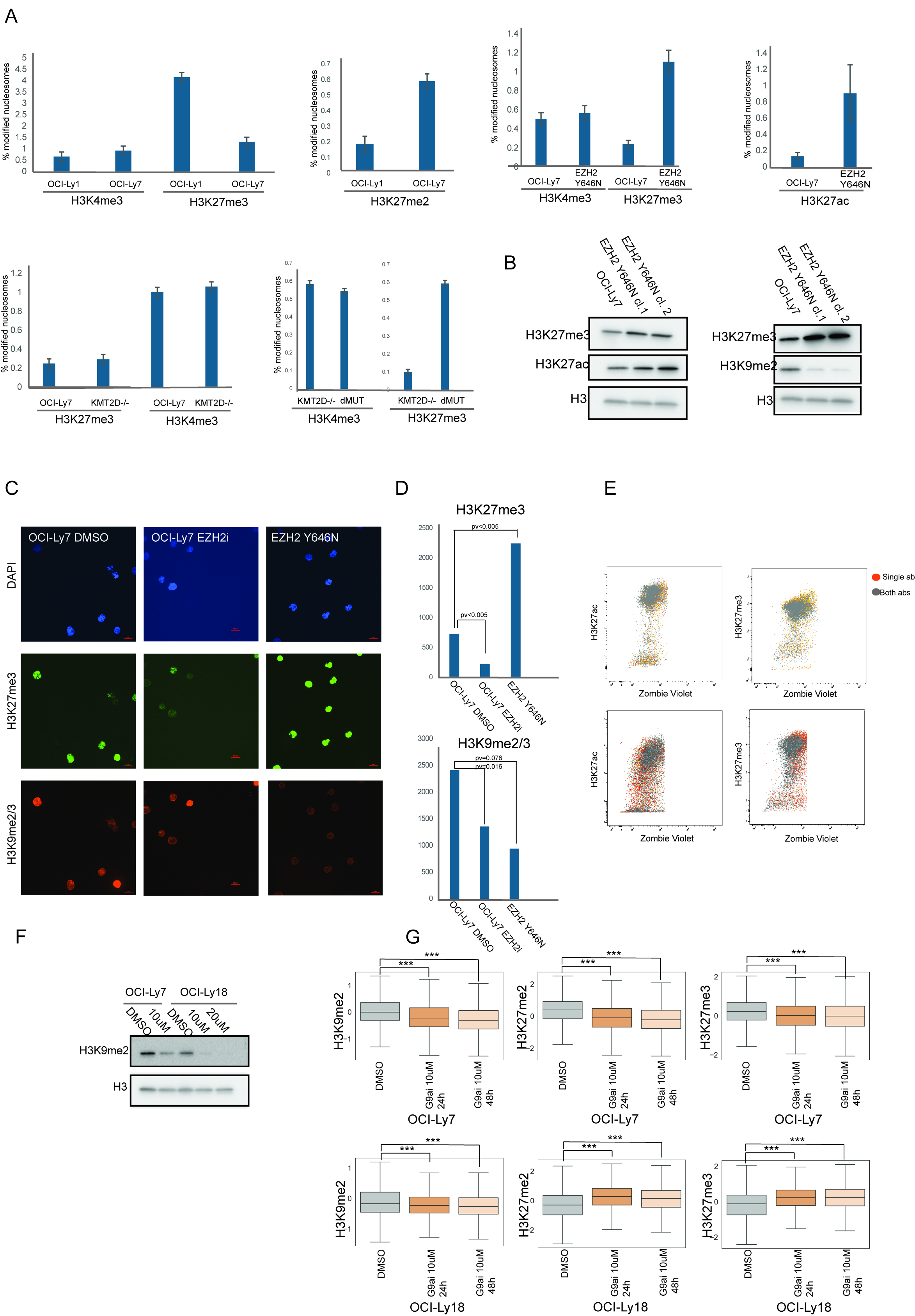

Supplement: S3 Fig — A. Single molecule analysis, as described in Furth and colleagues [87], of the percentage of modified nucleosomes for the indicated modification in the indicated sample. Nucleosomes were extracted using MNase digestion and tagged with a fluorophore-biotin conjugate, then captured on a streptavidin-coated surface. Next, nucleosomes were incubated with fluorescently-labeled antibodies targeting the indicated modifications. Values represent colocalization events of antibody signal with nucleosome signal, measured via TIRF microscopy. Full list of all antibodies is depicted in S2 Table. Results validate the CyTOF analysis in Fig 1G. B. Western blot analysis of the indicated modifications in the isogenic OCI-Ly7 EZH2 WT and EZH2 Y646N cells. Histone H3 represents loading control. Results validate the CyTOF analysis in Fig 1G. C–D. Immunofluorescence of OCI-Ly7 and EZH2 Y646N cells for the indicated histone modifications (H3K9me2/3 AF-555 and H3K27me3 AF-488) and DAPI. EZH2 inhibitor was applied to OCI-Ly7 cells at a concentration of 10 µM for 48 h. EZH2 Y646N cells show robust reduction in H3K9me2/3 levels, compatible with the CyTOF analysis. Of note, H3K9me2/3 levels are detected here by a different antibody (clone and vendor) than the one used for CyTOF, providing an additional validation of the data. D. Mean values of the fluorescent signal (40–60 cells per sample) of the indicated histone modifications, as seen in S3C Fig. P values were calculated by T test. E. Two independent repeats of FACS analysis of OCI-Ly7 cells, stained with fluorescent H3K27me3 and H3K27ac antibodies. Scatterplots represent antibody signal against Zombie Violet dye of samples stained with one antibody only, or both antibodies together. The results indicate that there is no apparent interference between the antibodies, as the signals of staining the cells with a single antibody or a combination of both are comparable. F. Western blot analysis of H3K9me2 in OCI-Ly7 or OCI-Ly18 cells. Cells were [file pbio.3003191.s003.tif]

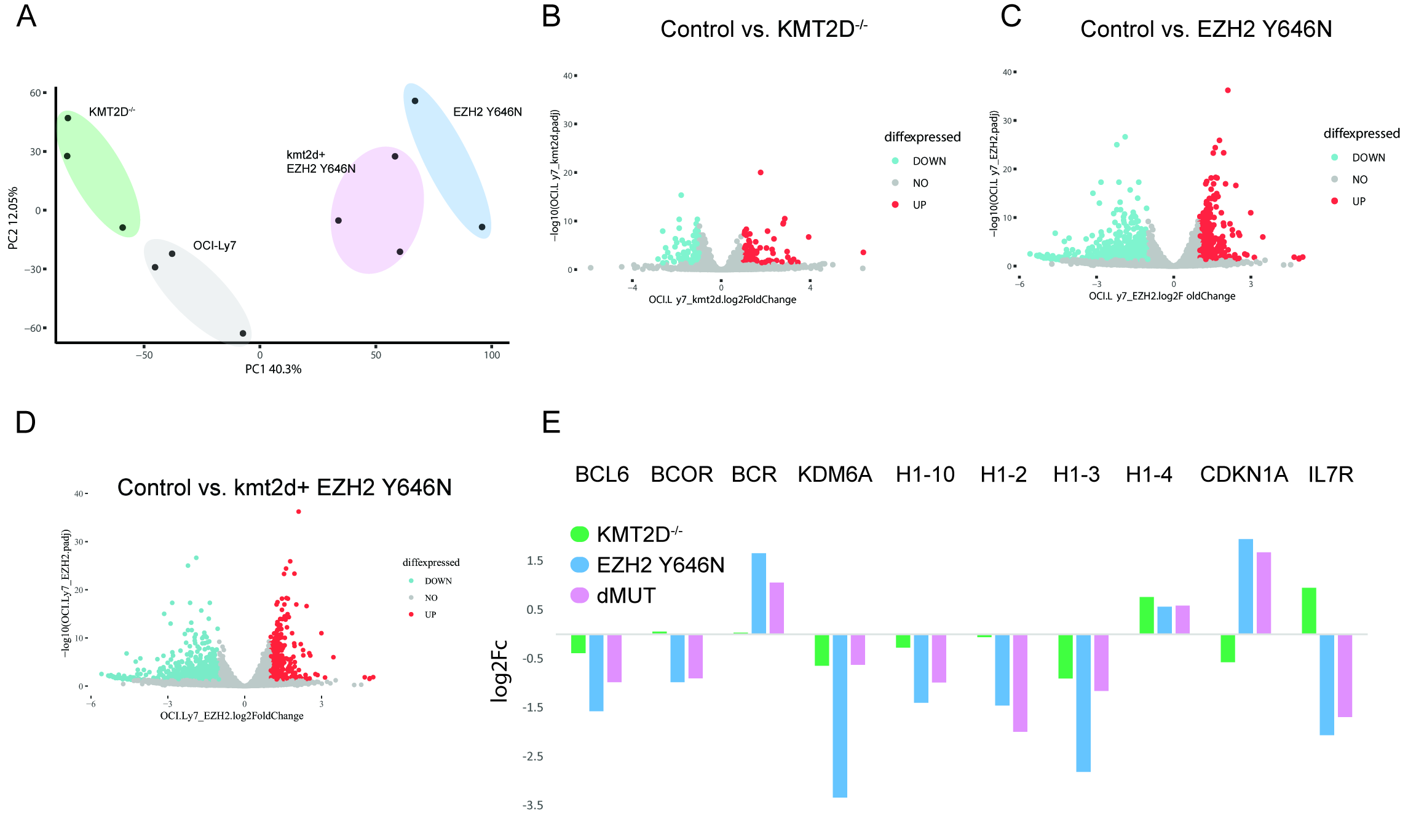

Supplement: S4 Fig — A. RNA-sequencing analysis of OCI-Ly7 cells and the indicated isogenic mutant lines. Principal component analysis of count number for all genes. Showing PC1 (40.3%) and PC2 (12.05%). B–D. Volcano plots showing Log fold change and p.adj values of differentially expressed genes between the indicated mutant lines versus OCI-Ly7. The list of differentially expressed genes was obtained using DESeq2. Upregulated genes marked in blue were filtered by >1 LogFC and p.adj ≤ 0.05. Downregulated genes marked in red were filtered by <−1 LogFC and p.adj ≤ 0.05. E. Visualization of OCI-Ly7 H3K27me3 read coverage around the TSS, as measured by Cut&Run, of differential genes from clusters 2 and 4 (Fig 1H). F. Bar plot showing Log2 change values of the indicated genes in the mutant cell lines versus OCI-Ly7. Colors indicate sample index. The data underlying this figure can be found in Raw data 1, 3 at 10.17605/OSF.IO/NTGUX, under CyTOF and Cut and Run folders. (TIF) [file pbio.3003191.s004.tif]

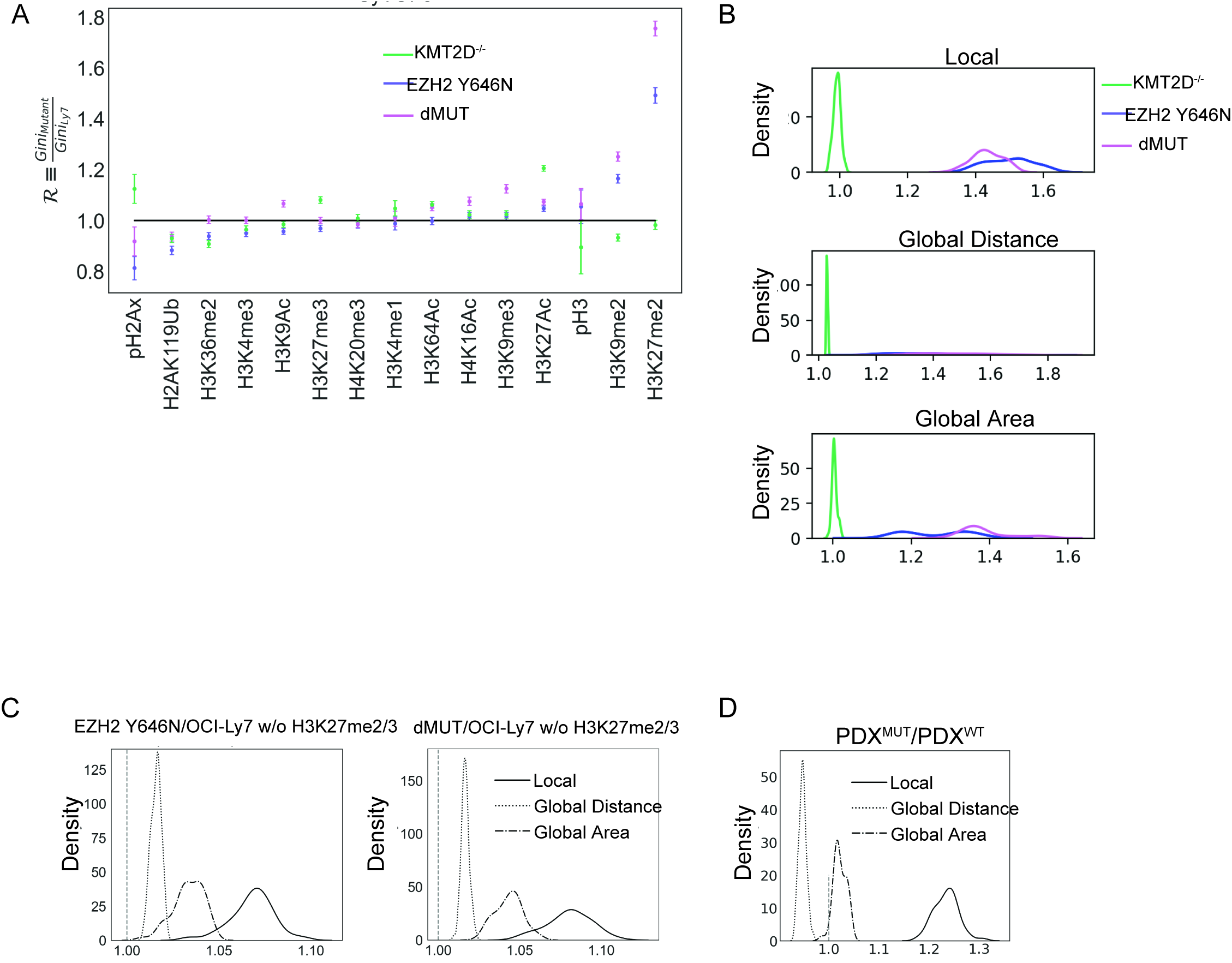

Supplement: S5 Fig — A. The Gini coefficient, commonly used to measure the inequality among the values of a frequency distribution, was used to determine the relative heterogeneity of each modification between the different cell lines. The ratio of the Gini coefficient between the individual lines and the WT was used as a measure of the excess heterogeneity of the mutant over the WT for each modification. B. The indicated heterogeneity measurements that were defined in Fig 2C were calculated for OCI-Ly7 cells versus each of the mutant lines, across a wide range of UMAP parameters. This experiment is a biological repeat of the experiment presented in Fig 2C. Plotted are the ratios of the values of each mutant line versus OCI-Ly7. Thus, values over 1 indicate excess heterogeneity in the mutant over that of the WT. EZH2 mutant cells showed higher heterogeneity in all measurements. C. Heterogeneity measurements calculated for OCI-Ly7 versus the GOF cells or the double mutant with KMT2D. Values were calculated based on the UMAPs presented in S2E Fig, without H3K27me2/3. EZH2-mutant cells show higher heterogeneity locally and globally, even when removing H3K27me2/3 from the analysis. D. Heterogeneity measurements calculated for the two patient derived xenografts with WT EZH2 (PDXWT) versus mutant-EZH2 (PDXMUT). Overall, EZH2-mutant cells show higher heterogeneity locally, and for global area. The data underlying this figure can be found in Raw data 1 at 10.17605/OSF.IO/NTGUX, under CyTOF folder and S1 Raw images. (TIF) [file pbio.3003191.s005.tif]

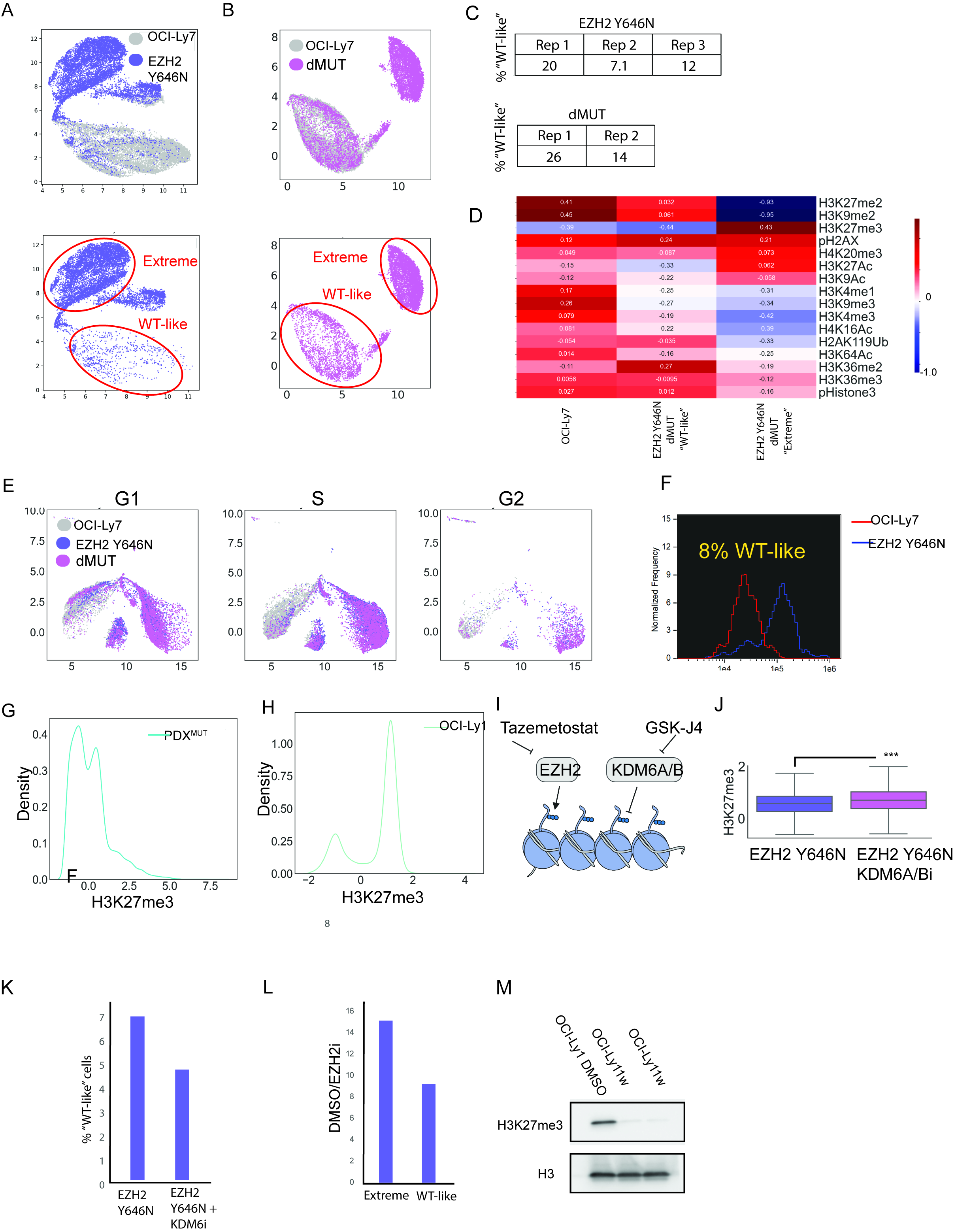

Supplement: S6 Fig — A–B. Top: OCI-Ly7 cells expressing WT EZH2, mutant-EZH2 (EZH2 Y646N), or a combination of mutant-EZH2 with KMT2D biallelic knockout (dMUT) were analyzed by CyTOF. Shown are joint UMAPs of OCI-Ly7 cells (WT) and the indicated lines. A represents a repeat of the experiment shown in Fig 2F. B shows two repeats for the double mutant (KMT2D−/− EZH2 Y646N). Colors indicate the sample index. Bottom: Only mutant-EZH2 cells are plotted, to highlight the subpopulation of mutant cells that cluster with WT cells, referred to as ‘WT-like’. C. The fraction of WT-like cells in three biological CyTOF replicates for the single EZH2 Y6464 mutant line, and two biological replicates for the double mutant. The percentage of cells consisting of this subpopulation is dynamic and varies between experiments. D. The mean of distribution of the indicated histone modifications for OCI-Ly7 cells expressing WT EZH2 (‘WT’), and for the double mutant of EZH2 Y646N with KMT2D knockout (dMUT) cells that either express robustly the GOF phenotype and form a distinct cluster (EZH2 Y646N dMUT ‘Extreme’), or the ‘WT-like’ subpopulation that clusters with OCI-Ly7 WT cells. See also Fig 2G for the single EZH2 mutant line. E. OCI-Ly7 cells and the indicated isogenic mutants were analyzed by CyTOF, that included all epigenetic modifications as well as the Maxpar cell cycle panel kit. The cell cycle markers, included in the CyTOF panel, were used to determine the cell cycle phase of each cell: G1, S and G2. For each phase, a joint UMAP of the WT and mutant cells was generated, based on all epigenetic modifications. ‘WT-like’ cells are observed for each of the indicated cell cycle phases. F. Histogram showing image stream values of H3K27me3 in OCI-Ly7 and EZH2 Y646N. In this complementary technology to CyTOF, we also observed a bimodal distribution for H3K27me3, indicating a subpopulation of WT-like cells (8% of the EZH2 Y646N cells). G–H. Histogram of H3K27me3 levels in: G. Patient-derived xenograft expressin [file pbio.3003191.s006.tif]

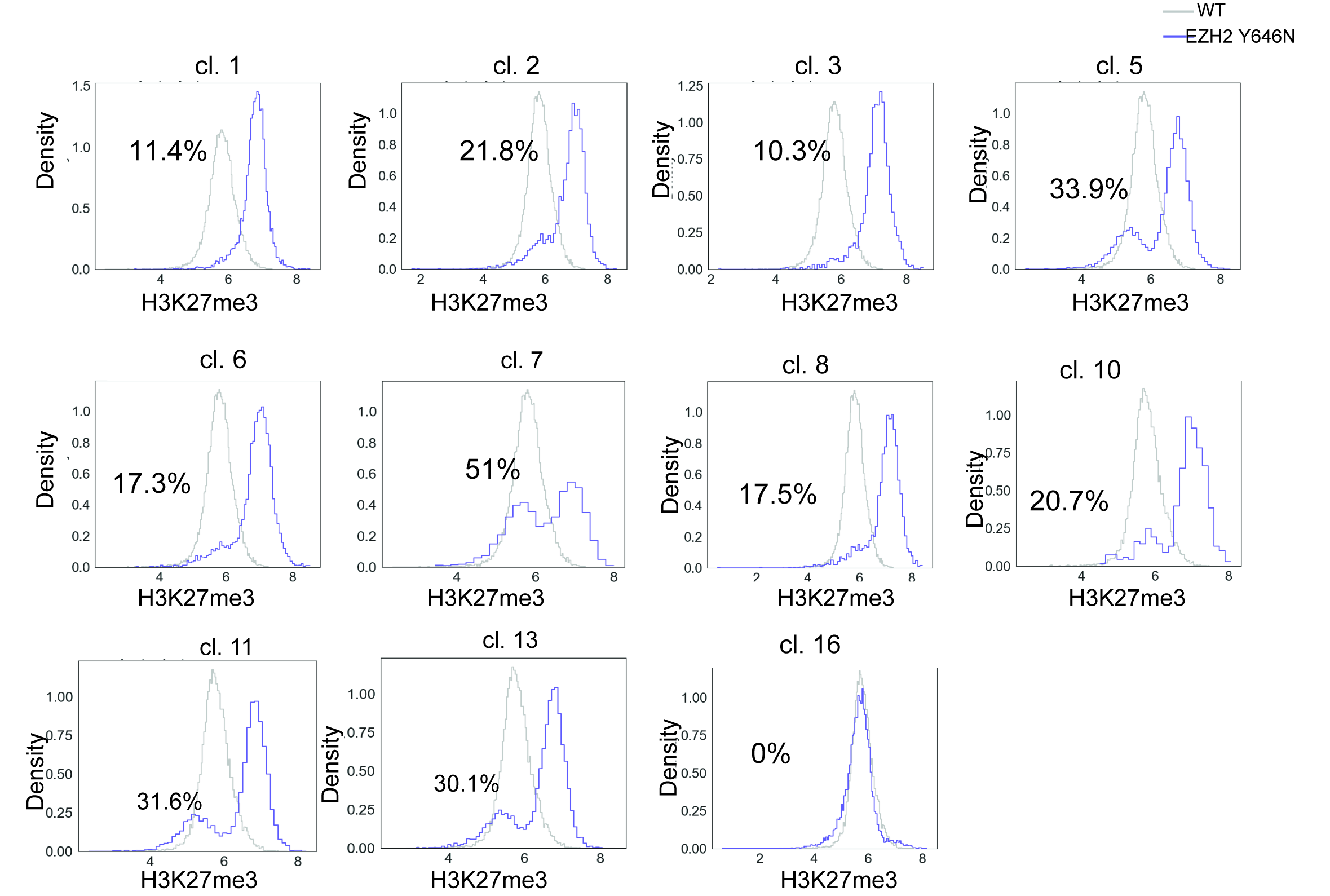

Supplement: S7 Fig — Histogram of scaled and normalized H3K27me3 levels, as measured by CyTOF, in OCI-Ly7 cells (WT) or the indicated EZH2 Y646N single-cell clones. The percentage of ‘WT-like’ cells is shown per clone. The data underlying this figure can be found in Raw data 1 at 10.17605/OSF.IO/NTGUX, under CyTOF folder. (TIF) [file pbio.3003191.s007.tif]

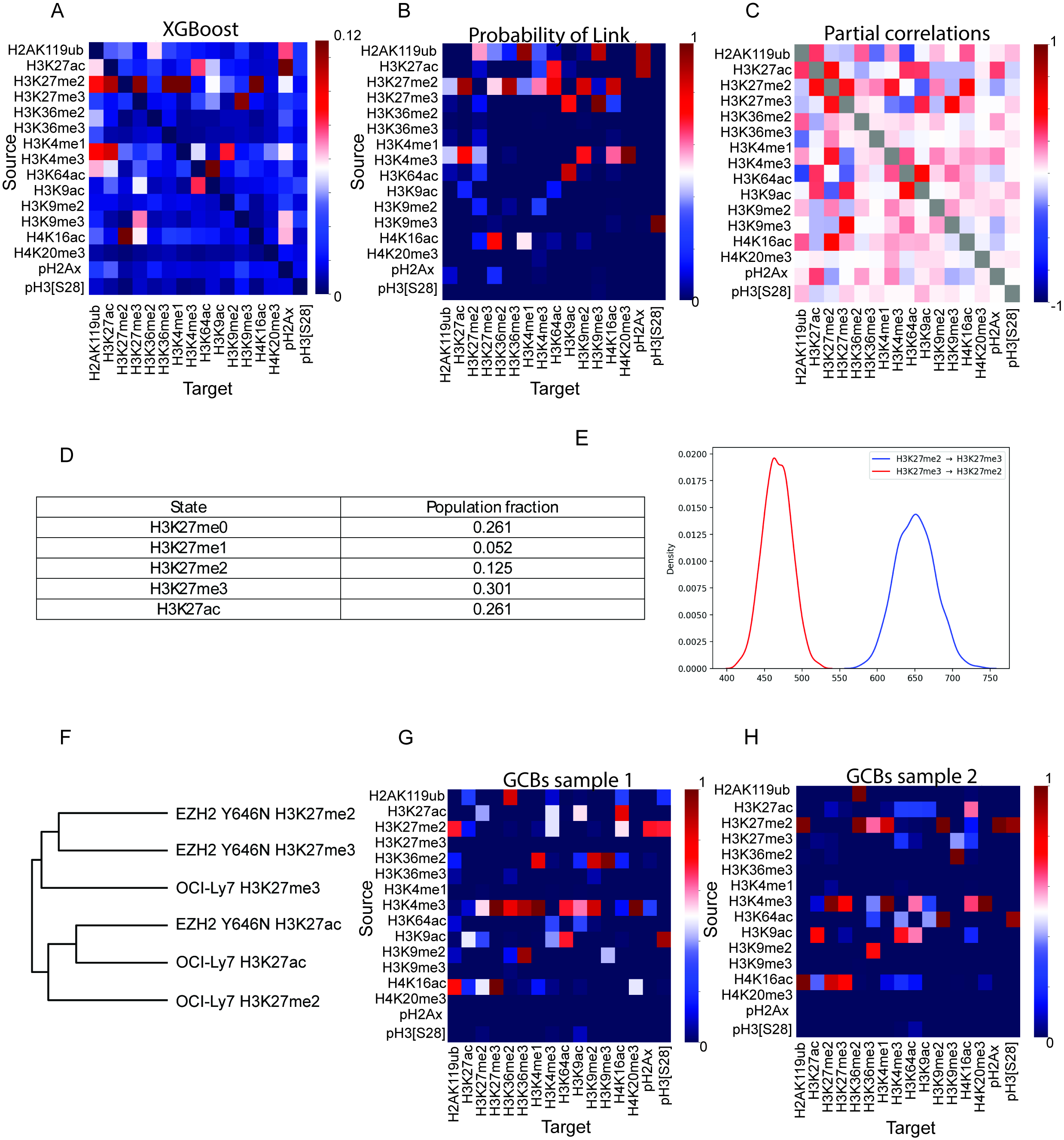

Supplement: S8 Fig — A–C. Models to decipher interactions within the epigenetic network, applied to unperturbed OCI-Ly7 single-cell CyTOF data, on a biological repeat of the experiment shown in Fig 3A–C. A. XGBoost analysis. B. ‘Probability of link’ analysis. For both A and B, the Y axis indicates ‘source’ modification and X axis indicates its ‘target’ modification affected by the Y axis. C. Partial correlations between histone modifications. D–E. Markov chain simulation of the H3K27 methylation and acetylation dynamics (See “Methods”). D. The table shows the steady-state populations in our simulation, representing the fraction of nucleosomes modified within a cell. E. Shown are the distribution of the SHAP values, associated with the effect of H3K27me2 on H3K27me3 (blue) or H3K27me3 on H3K27me2 (red). The distribution shown is for 500 runs over a subsample of the data in order to allow for the statistical variation. As our model predicts, there is a stronger effect of H3K27me2 on H3K27me3 compared to the reverse direction. F. Dendrogram of the hierarchical clustering of spearman correlations of Cut&Run normalized reads between all samples, calculated on 10kbp genomic bins. G–H. Probability of link analysis done on CyTOF data of unperturbed germinal-center B cells (CD20+, CD38+), derived from tonsils, for two independent samples derived from different patients. Tonsils were dissociated to single cells followed by staining with the panel of metal-conjugated antibodies and CyTOF analysis. The data underlying this figure can be found in Raw data 1, 3 at 10.17605/OSF.IO/NTGUX, under CyTOF and Cut and Run folders and S1 Raw images. (TIF) [file pbio.3003191.s008.tif]

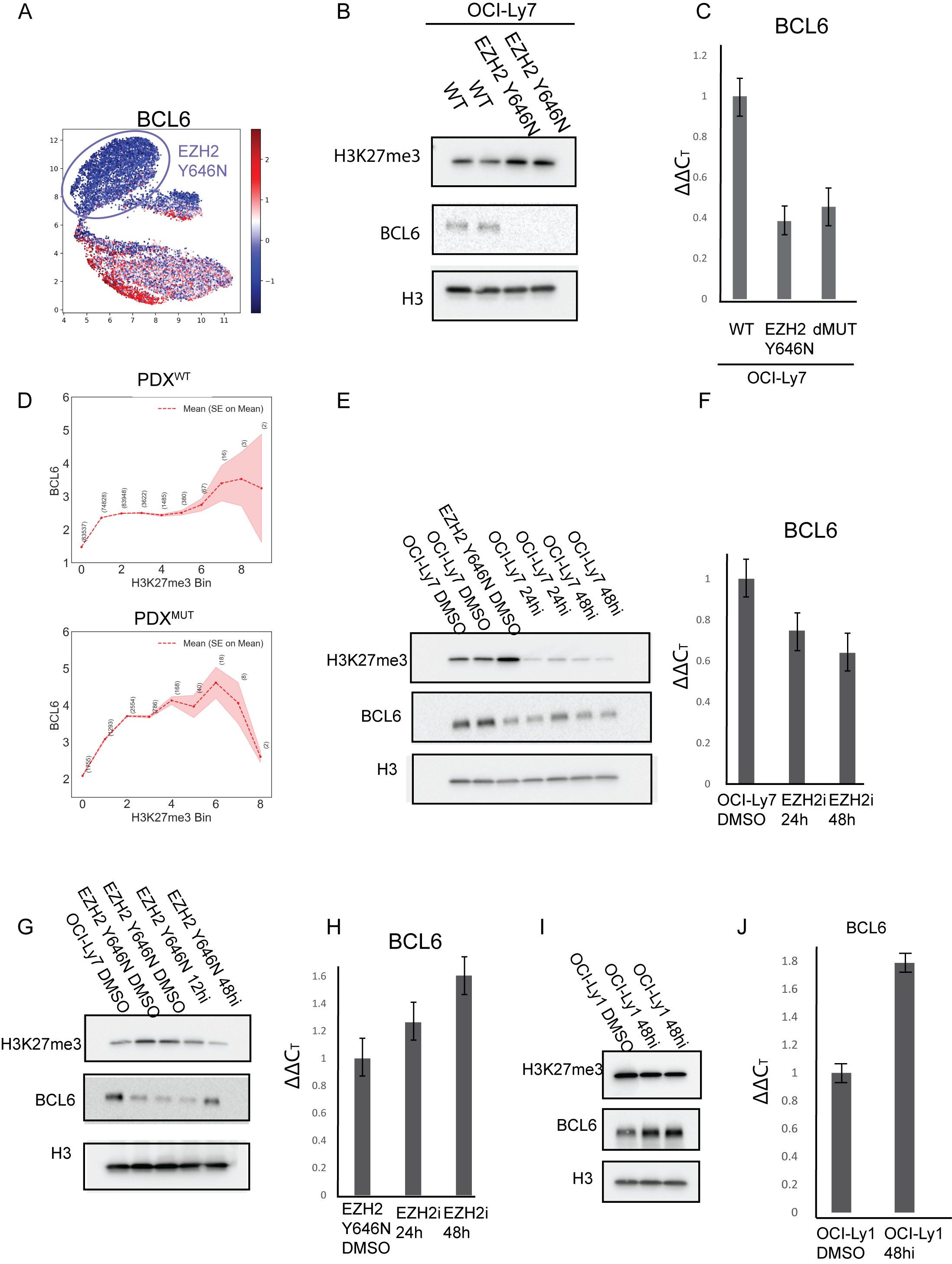

Supplement: S9 Fig — A. Scaled, normalized levels of BCL6 on the joint UMAP of OCI-Ly7 and EZH2 Y646N cells, corresponding to the UMAP shown in Fig 2A. Cells expressing mutant-EZH2 show downregulation of BCL6 levels. B. Western blot analysis of H3K27me3 and BCL6 in the indicated samples. Histone H3 is used as a loading control. C. Quantitative RT-PCR analysis of BCL6 expression in the isogenic OCI-Ly7 WT and EZH2-mutant cells. ΔΔCT values relative to OCI-Ly7 ±s.d (n = 3) are shown. HPRT was used for normalization. D. BCL6 mean levels in cells binned according to H3K27me3 levels. Red hue represents standard error of the mean. Number of cells in each bin is shown. Top: PDX with WT-EZH2 (PDXWT). Bottom: PDX with mutant-EZH2 (PDXMUT). H3K27me3 and BCL6 show non-linear relationship. E–F. Western blot analysis of H3K27me3 and BCL6. Histone H3 is used as a loading control. E. OCI-Ly7 cells were treated with EZH2 inhibitor at a concentration of 10 µM for the indicated times. F. EZH2-mutant cells were treated with EZH2 inhibitor at a concentration of 10 µM for 48 h. G–H. Quantitative RT-PCR analysis of BCL6 expression in the isogenic OCI-Ly7 WT and EZH2-mutant cells, treated with EZH2i 10 µM at the indicated times. ΔΔCT values relative to OCI-Ly7 ±s.d (n = 3) are shown. HPRT was used for normalization. I. Western blot analysis of H3K27me3 and BCL6. OCI-Ly1 cells, carrying mutant-EZH2, were treated with EZH2 inhibitor at a concentration of 10 µM for 48 h. J. Quantitative RT-PCR analysis of BCL6 expression in OCI-Ly1 cells treated with EZH2 inhibitor at a concentration of 10 µM for 48 h versus DMSO control. ΔΔCT values relative to DMSO treated sample ±s.d (n = 3) are shown. The data underlying this figure can be found in Raw data 1, 6 at 10.17605/OSF.IO/NTGUX, under CyTOF and RT-PCR folders and S1 Raw images. (TIF) [file pbio.3003191.s009.tif]
