# Supplementary material for: Mutant EZH2 alters the epigenetic network and increases epigenetic heterogeneity in B cell lymphoma
Source: PLoS Biol. 2025 Jun 12;23(6):e3003191. doi: 10.1371/journal.pbio.3003191 (PMC12161531; doi:10.1371/journal.pbio.3003191)

S2B

H3K27me3

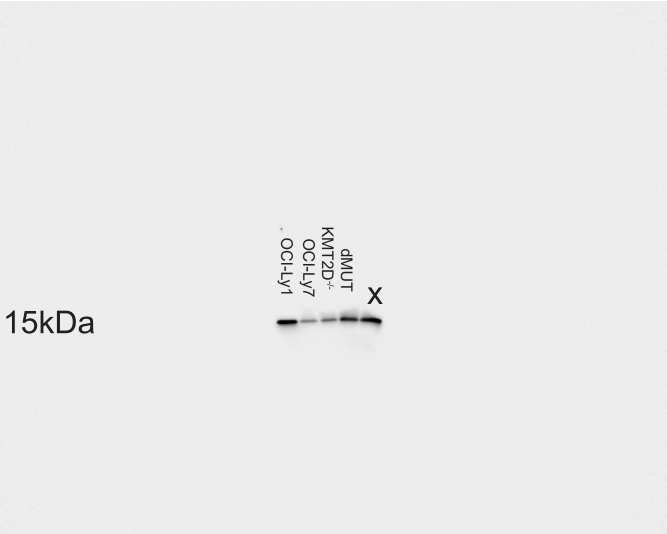

H3

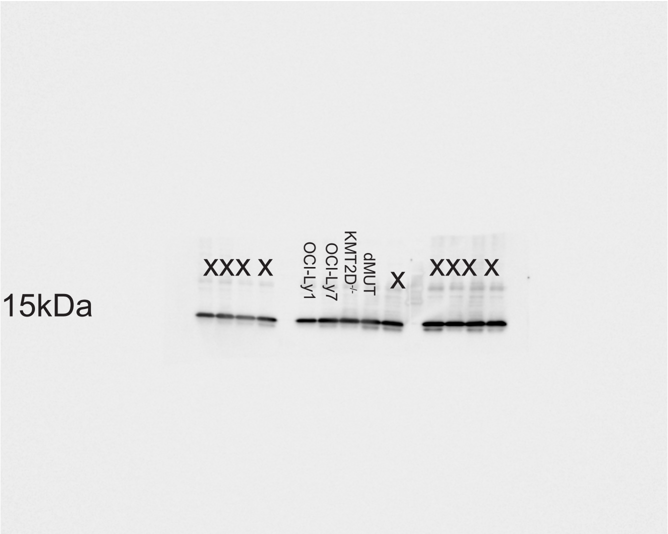

S2C

H3K27me3

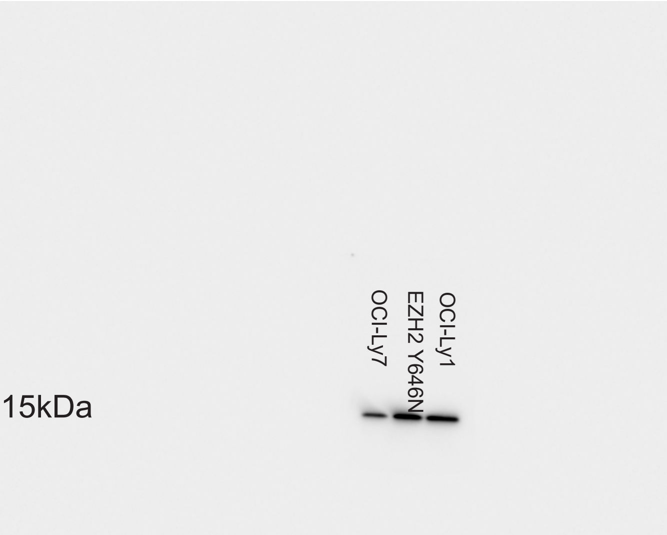

H3

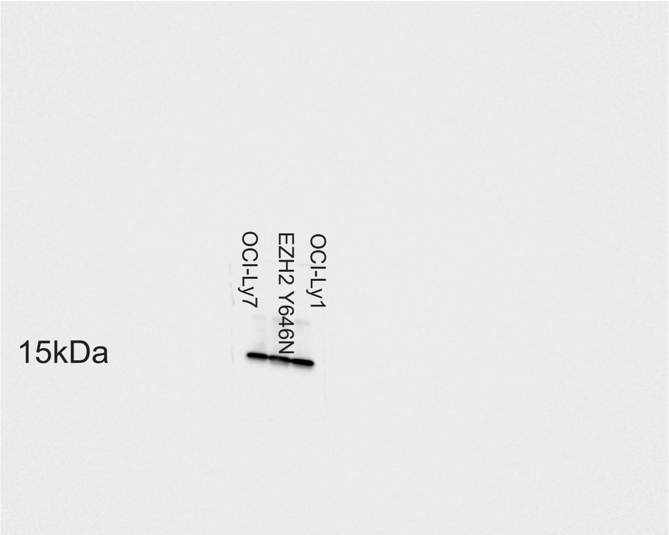

H3K27me2

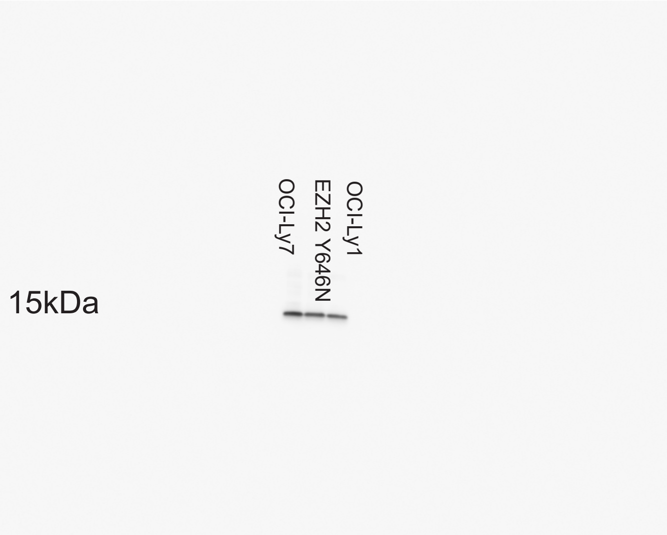

S3B

Left

H3

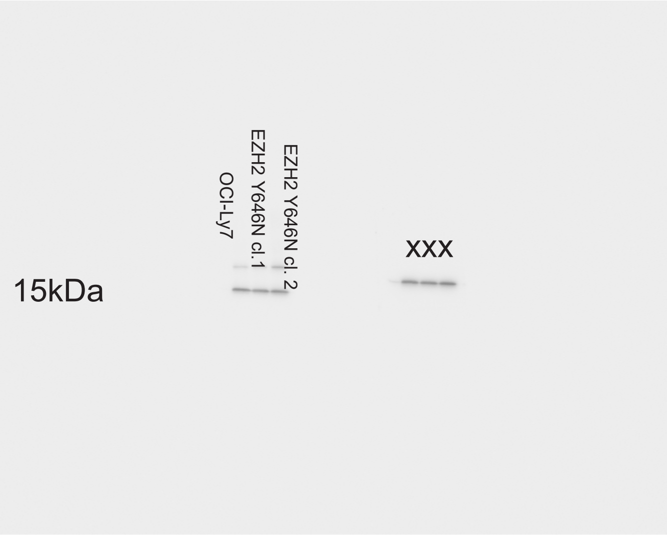

H3K27me3

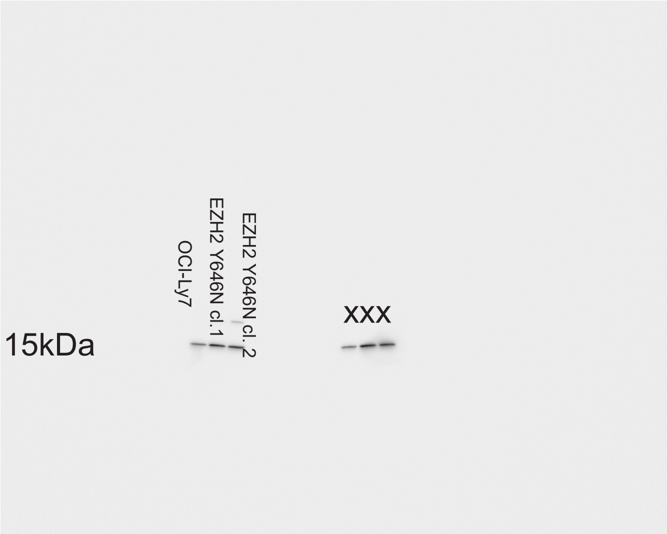

H3K27ac

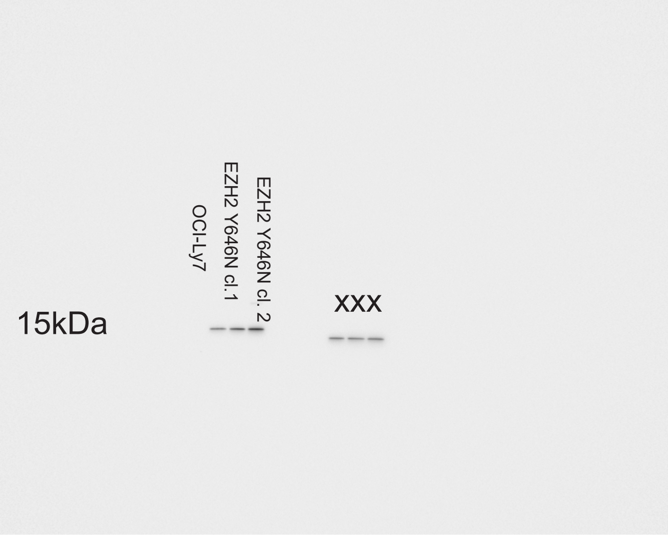

Right

H3K27me3

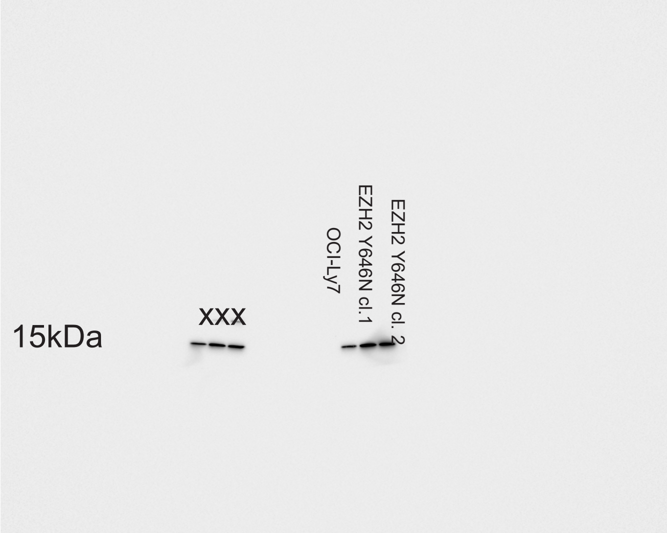

H3K9me2

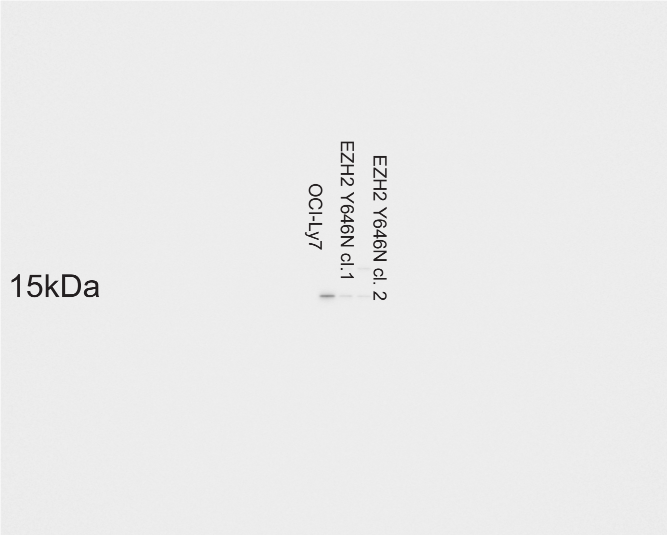

H3

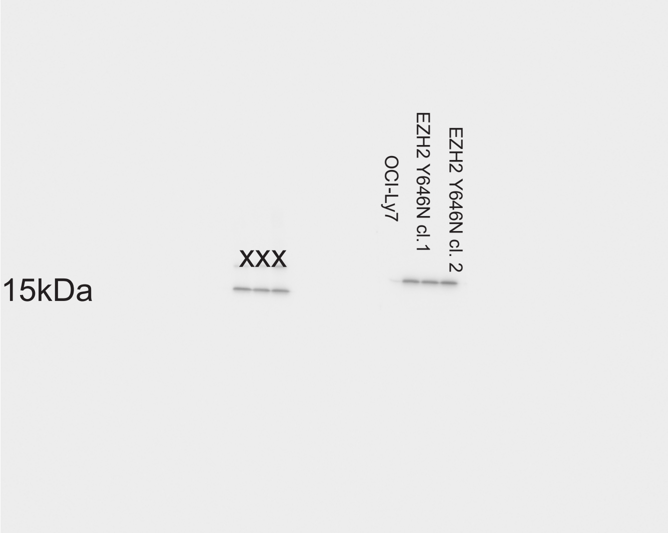

S3F

H3

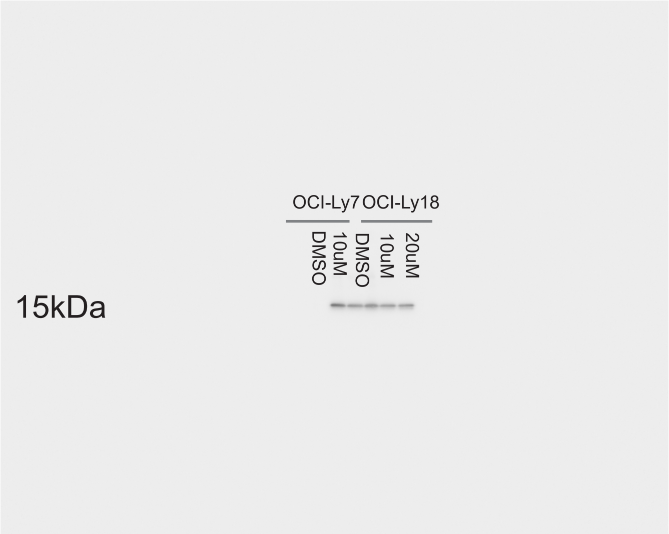

H3K9me2

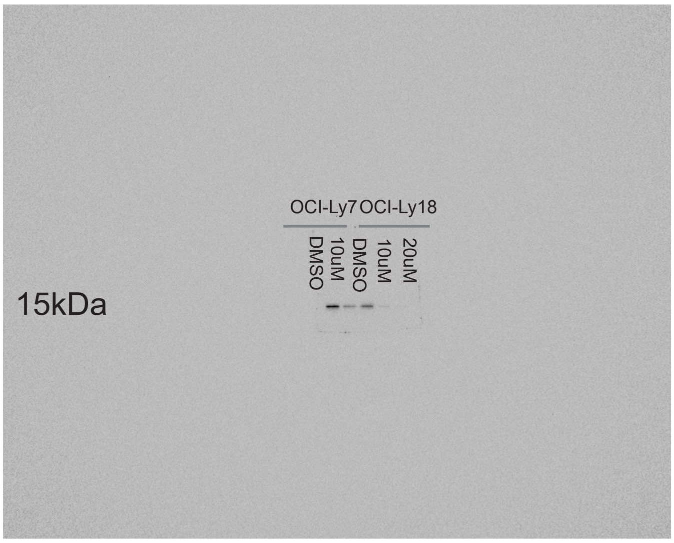

S6M

H3

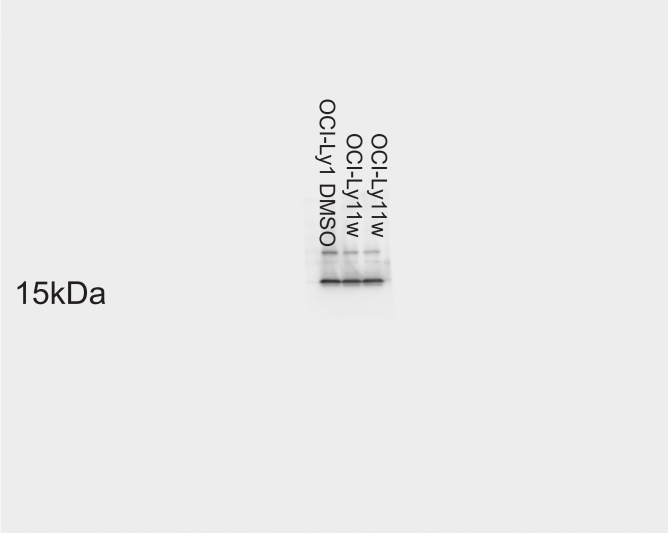

H3K27me3

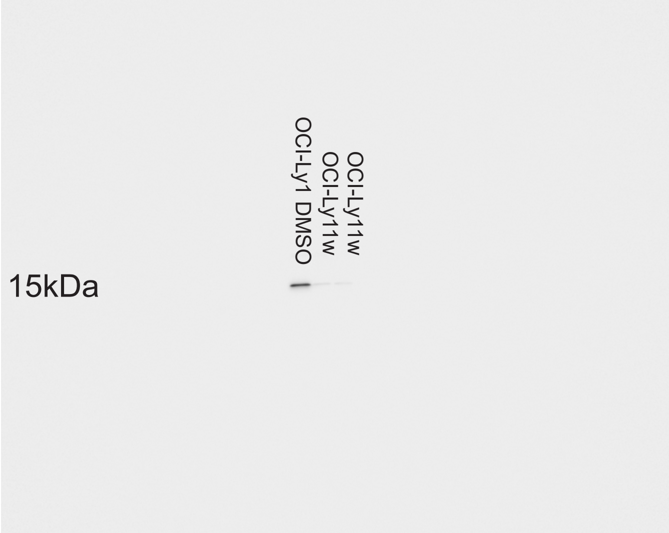

S9B

H3

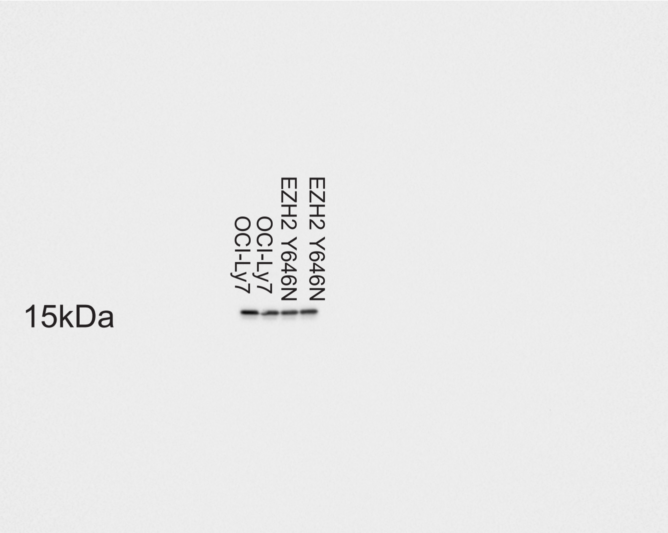

BCL6

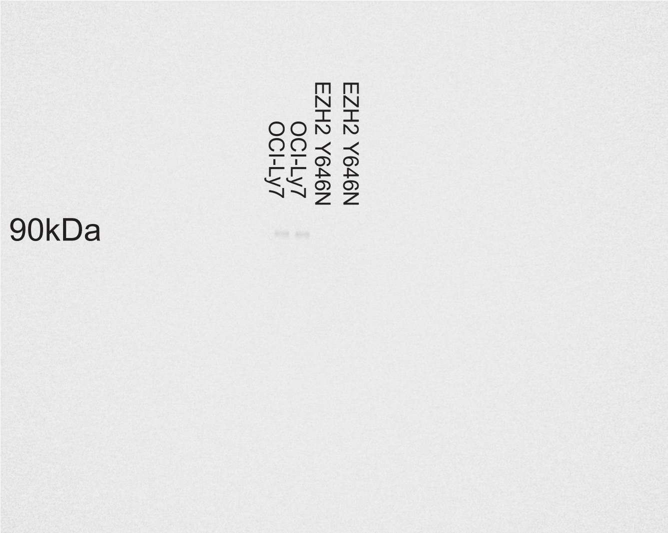

H3K27me3

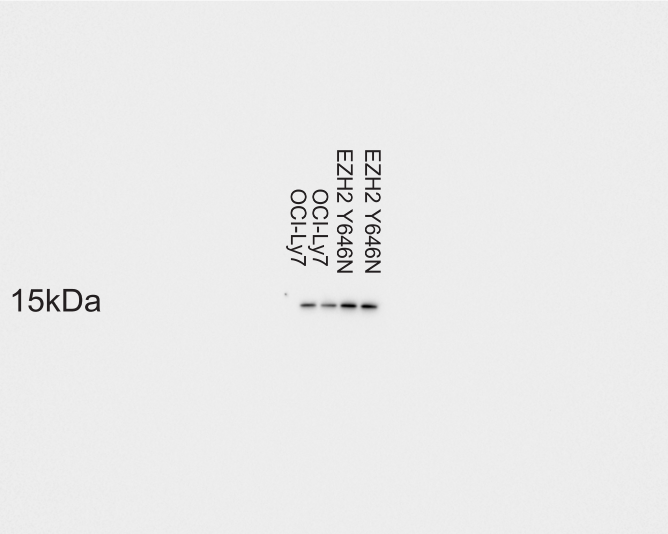

S9E

H3K27me3

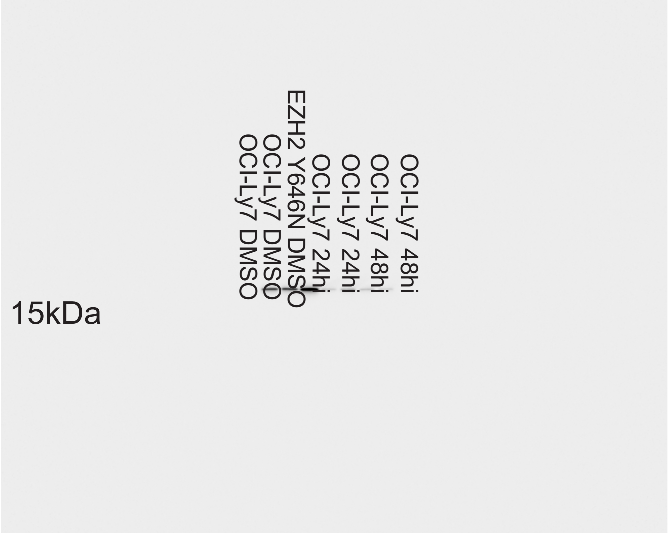

BCL6

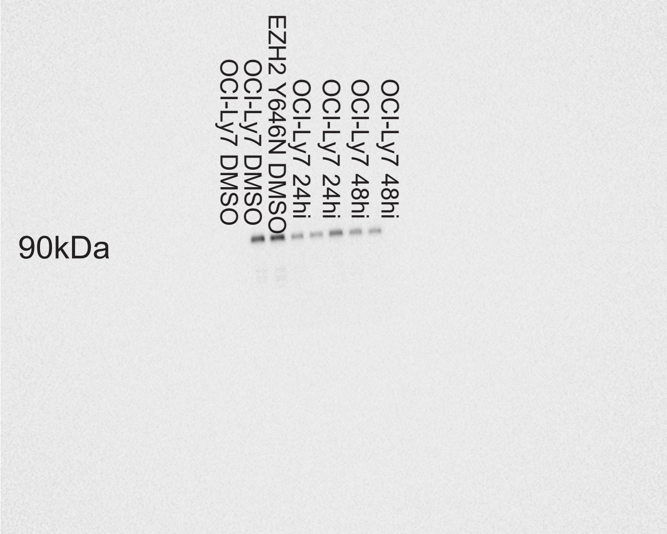

H3

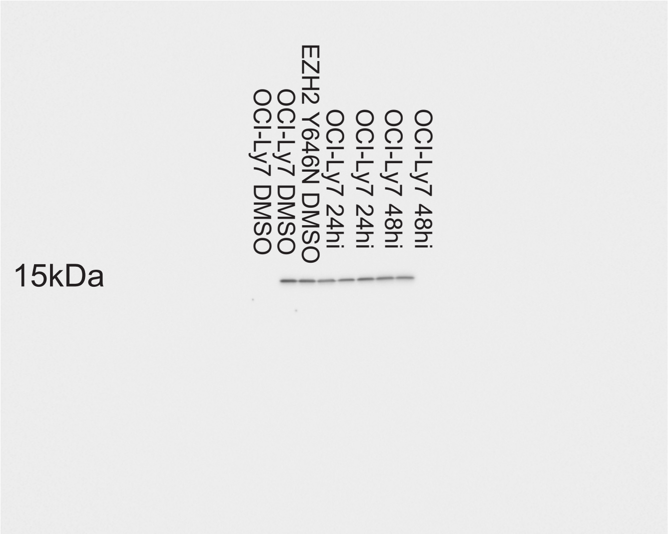

S9G

H3K27me3

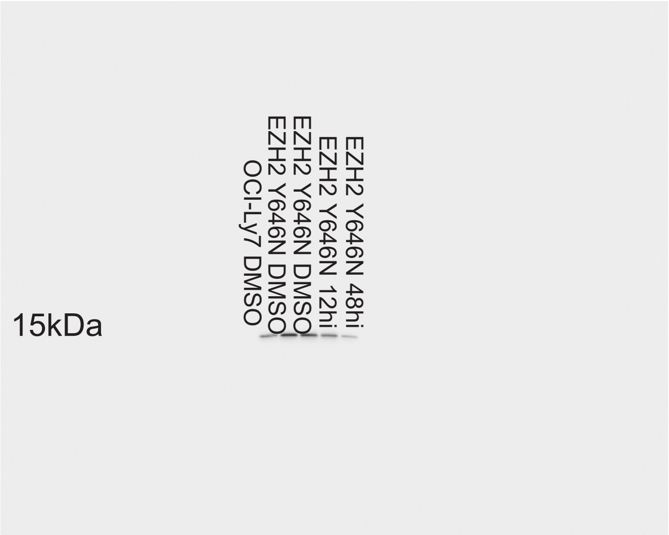

BCL6

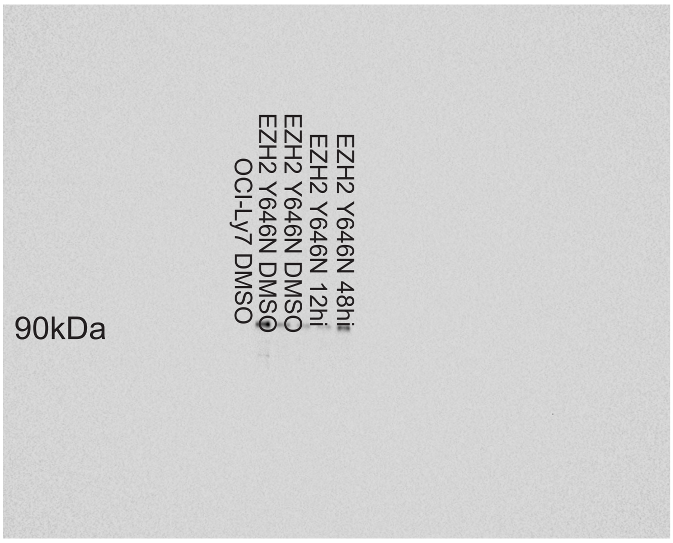

H3

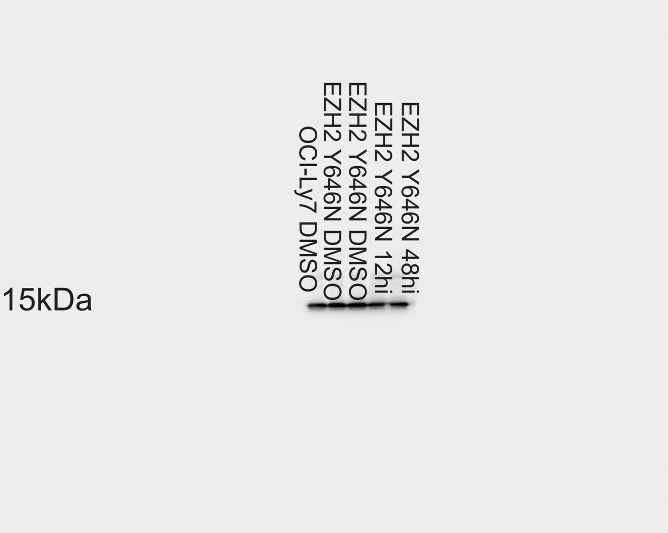

S9I

H3K27me3

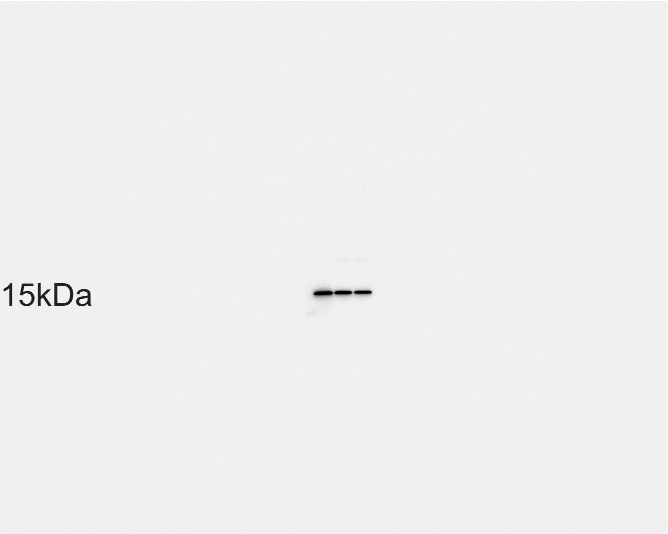

BCL6

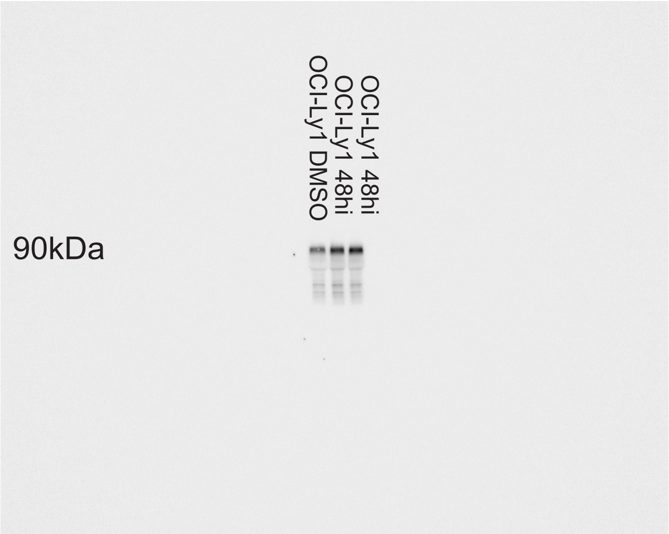

H3

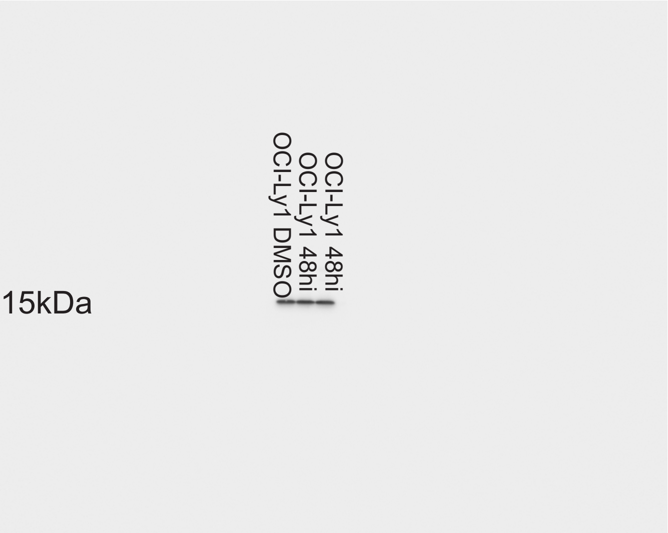

Supplement: S1 Raw images — Raw uncropped images of Western Blotting in this study for S2B, S2C; S3B, S3F; S6M, S9B, S9E, S9G and S9I Figs. Doi: 10.6084/m9.figshare.28737236. (PDF) [file pbio.3003191.s012.pdf]
